# Study on the mechanism of hinokitiol in inhibiting the biofilm activity of *Staphylococcus epidermidis*

**DOI:** 10.3389/fmicb.2026.1811623

**Published:** 2026-05-21

**Authors:** Ting Zhang, Junhao Xiang, Na Yu, Yaoxin Tang, Jing Qu, Yiming Liu, Xiubo Li

**Affiliations:** 1 National Feed Drug Reference Laboratories, Feed Research Institute, Chinese Academy of Agricultural Sciences, Beijing, China; 2 Key Laboratory of Animal Antimicrobial Resistance Surveillance, Ministry of Agriculture and Rural Affairs, Feed Research Institute, Chinese Academy of Agricultural Sciences, Beijing, China; 3 Laboratory of Quality and Safety Risk Assessment for Products on Feed-Origin Risk Factor, Ministry of Agriculture and Rural Affairs, Feed Research Institute, Chinese Academy of Agricultural Sciences, Beijing, China

**Keywords:** antibacterial mechanism, biofilm, hinokitiol, mastitis, quorum sensing, *Staphylococcus epidermidis*

## Abstract

Mastitis in dairy cows is one of the most prevalent infectious diseases in the dairy industry, leading to reduced milk production, increased production costs, and substantial economic losses. Antibiotics are commonly used to prevent and treat mastitis; however, persistent biofilm formation by *Staphylococcus epidermidis* (*S. epidermidis*) has contributed to rising antibiotic resistance and poses significant challenges for complete eradication. Therefore, the development of effective therapeutic strategies is urgently needed. This study investigated the antibacterial and anti-biofilm activities of hinokitiol, both alone and in combination with Ceftiofur sodium, against biofilm-producing *S. epidermidis*. The checkerboard assay revealed a significant synergistic effect between hinokitiol and Ceftiofur sodium (FICI ≤ 0.5). Importantly, hinokitiol exhibited negligible cytotoxicity toward mammalian cells and low hemolytic activity against sheep erythrocytes at therapeutic concentrations. Mechanism studies demonstrated that hinokitiol disrupted bacterial membrane permeability, as evidenced by increased NPN uptake, PI staining, and significant intracellular protein leakage. Furthermore, hinokitiol induced the accumulation of intracellular reactive oxygen species (ROS), leading to oxidative stress-mediated cell death. Transcriptomic analysis and RT-qPCR validation showed that hinokitiol significantly down-regulated the expression of key genes involved in biofilm formation (*icaA, fnbA, aap*), quorum sensing (*agrA, luxS*), and global regulation (*sarA, sigB*), while up-regulating the repressor *icaR*. These findings suggest that hinokitiol exerts its antibacterial and anti-biofilm effects by damaging the cell membrane, inducing oxidative stress, and modulating multi-target regulatory pathways, making it a promising candidate for treating *S. epidermidis* infections. However, this study is limited by the absence of *in vivo* mastitis models and pharmacokinetic data. Future research should focus on validating these findings in animal models, optimizing administration routes (e.g., intramammary formulations), and assessing clinical efficacy in field conditions to facilitate the translation of hinokitiol into a viable therapeutic option for refractory *S. epidermidis* mastitis.

## 1 Introduction

Bovine mastitis is the most prevalent infectious disease with the most severe economic losses in the dairy industry, posing a serious threat to global dairy safety and animal welfare. *Staphylococcus* is a zoonotic pathogenic bacterium that can be classified into coagulase-positive and coagulase-negative categories. For a long time, it was believed that only coagulase-positive staphylococci, specifically *Staphylococcus aureus*, could cause severe infections, resulting in relatively limited research on coagulase-negative staphylococci (CNS). However, with the advancement of medicine, the accelerated use of interventional therapies, broad-spectrum antibiotics, and immunosuppressive agents has led to CNS becoming an important pathogen in hospital-acquired infections, endangering human lives and continuously drawing attention. CNS can adhere to host cell surfaces, with bacterial cells aggregating to form biofilms, thereby preventing neutrophil phagocytosis and antibiotic penetration. CNS can cause infections in various human tissues, including the urinary tract, skin, soft tissues, and endocardium.

Coagulase-negative *staphylococci* (CNS) are among the most prevalent bacteria detected in bovine milk samples across multiple regions worldwide. Certain CNS species exert a more significant impact on udder health than other *staphylococci*. CNS primarily causes subclinical mastitis in dairy cows, leading to persistent intramammary infections, with *Staphylococcus chromogenes*, *Staphylococcus haemolyticus* (*S. haemolyticus*) (Ajose et al., 2024), and *Staphylococcus epidermidis* (*S. epidermidis*) (Kløve et al., 2025) representing the predominant species. Due to the propensity of the CNS to form biofilms, antimicrobial resistance among these *staphylococci* is particularly severe (Nyman et al., 2018; Ajose et al., 2024). In recent years, Coagulase-negative *staphylococci,* exemplified by *Staphylococcus epidermidis*, have demonstrated a markedly increased detection rate in chronic and recurrent mastitis cases (Piessens et al., 2012; Qiu et al., 2025). *S. epidermidis* exhibits exceptional environmental adaptability and frequently establishes recalcitrant biofilms on the surfaces of the teat canal and mammary cistern, enabling the pathogen to evade host immune clearance and induce long-term subclinical infections (Kazemzadeh et al., 2025; Persson Waller et al., 2025). Owing to their strong tendency to form biofilms, CNS exhibit severe antimicrobial resistance within the genus *Staphylococcus*. The resistance profiles of CNS are fundamentally similar domestically and internationally, particularly regarding *β*-lactam antibiotics, where resistance has become extremely serious and warrants significant attention.

Natural bioactive molecules have become an important direction for the development of novel antimicrobial drugs due to their structural diversity, low toxicity, and multi-target antibacterial mechanisms. For example, flavonoids and terpenoids have shown great potential in modulating mammary inflammatory responses and inhibiting the virulence factors of pathogenic bacteria (Zacchino et al., 2017; Guimarães et al., 2019; Kong et al., 2025). As the primary components of plant essential oils, terpenoids can effectively penetrate bacterial lipid bilayers due to their exceptional hydrophobicity, leading to cell membrane dysfunction or interference with quorum-sensing (QS) systems (Tholl, 2015; Yamaguchi, 2022).

Hinokitiol is a natural monoterpenoid compound extracted from plants of the Cupressaceae family. Research has confirmed that hinokitiol possesses broad-spectrum antimicrobial, anti-inflammatory, and antioxidant activities. In studies concerning fungi, hinokitiol induces severe oxidative stress by chelating intracellular iron ions and disrupting the mitochondrial respiratory chain (Jin et al., 2021). Recent studies indicate that hinokitiol can induce lipid membrane expansion, providing evidence for its physical disruption of bacterial structures (Le et al., 2023; Wyżga et al., 2024). Although its antimicrobial potential has been preliminarily recognized, systematic research is still lacking regarding how hinokitiol interferes with the molecular regulatory networks governing *Staphylococcus epidermidis* biofilm formation, particularly its impact on the core regulatory gene icaR and the key quorum-sensing protein LuxS.

The present study aims to evaluate the antimicrobial and anti-biofilm activities of hinokitiol against both standard strains and clinical isolates of *S. epidermidis*. Furthermore, integrated transcriptomic and molecular biology approaches were employed to elucidate the underlying molecular mechanisms. Our findings are expected not only to offer a novel therapeutic candidate for the management of chronic bovine mastitis but also to provide a robust theoretical foundation for the application of natural terpenoids in combating bacterial biofilms.

## 2 Materials and methods

### 2.1 Strains and drugs

Hinokitiol, with a purity of 99.78% (CAS: 499–44-5), was purchased from Shanghai Aladdin Biochemical Technology Co. A stock solution of Hinokitiol was prepared at a concentration of 20 mg/mL in [Solvent, dimethyl sulfoxide (DMSO) or ethanol] and stored at −20 °C until use. Clinical isolated strain S1, *S. aureus,* and *S. haemolyticus*; *Streptococcus agalactiae* (ATCC 13813); *Staphylococcus lugdunensis* (ATCC 49576); *S. lugdunensis* (ATCC-49576); *S. epidermidis* ATCC12228 (non-membrane-producing strain - standard strain), and *S. epidermidis* ATCC 35984 (membrane-producing strain) were purchased and preserved by our laboratory. Mammary Alveolar Cells-Large T antigen (MAC-T cells).

### 2.2 Main culture media and reagents

Luria-Bertani broth medium, Luria-Bertani Agar medium, Mueller-Hinton broth medium, and Mueller-Hinton Agar medium were all purchased from Qingdao Hope Biotechnology. Tryptone Soya Broth (TSB) and Tryptone Soy Agar (TSA) were purchased from Guangdong Huanca Biotechnology. PBS buffer (pH 7.2–7.4) was purchased from Beijing Solaibao Technology. Dimethyl sulfoxide (DMSO) and glycerol were purchased from Tianjin Damao Chemical Reagent Factory. Triton X-100 was purchased from Aladdin Bio. The BCA detection kit for detecting cell membrane permeability, the fluorescent dye propidium iodide (PI), the pH sensitivity probe BCECF-AM, the enhanced ATP detection kit, CCK8, and the reactive oxygen species (ROS) detection kit were purchased from Shanghai Beyotime Biotechnology, XTT.

### 2.3 Determination of minimum inhibitory concentration (MIC)

The MIC of hinokitiol against *S. epidermidis* was determined using the broth microdilution method in 96-well plates according to CLSI guidelines. Briefly, hinokitiol was dissolved and diluted in Mueller-Hinton Broth (MHB) to a stock concentration of 512 mg/L. To perform the assay, 100 μL of MHB was added to columns 2 to 11, and 200 μL of MHB was added to column 12 as a blank control. Subsequently, 200 μL of the hinokitiol solution was added to the first column, followed by a horizontal two-fold serial dilution from column 1 through column 10. Each well in columns 1 to 11 was then inoculated with 100 μL of the bacterial suspension (adjusted to approximately 1 × 10^6^ CFU/mL). Column 11, containing MHB and the inoculum without the drug, served as the growth control. After incubation at 37 °C for 18–24 h, the MIC was defined as the lowest concentration of hinokitiol that inhibited visible bacterial growth.

### 2.4 Growth inhibition curve

To evaluate the dynamic antibacterial effect of hinokitiol, growth curves were constructed at various concentrations (2 × MIC, MIC, and 0.5 × MIC), with a drug-free group serving as the growth control. Briefly, hinokitiol and the bacterial inoculum were prepared in Cation-Adjusted Mueller-Hinton Broth (CAMHB). The bacterial suspension was adjusted to a final density of approximately 1 × 10^6^ CFU/mL in the presence of hinokitiol at the specified concentrations. The mixtures were then incubated at 37 °C with constant shaking. The optical density at 600 nm was monitored at specific time intervals (0, 2, 4, 6, 8, 12, and 24 h) using a spectrophotometer. All experiments were performed in triplicate, and the growth curves were plotted as OD_600nm_ versus time.

### 2.5 Hemolytic activity assay

The hemolytic potential of hinokitiol was evaluated using sterile defibrinated sheep blood. Briefly, the red blood cells (RBCs) were harvested by centrifugation at 800 × g for 5 min, washed three times with phosphate-buffered saline (PBS), and resuspended to a final concentration of 5%. hinokitiol was serially diluted in PBS to achieve a range of concentrations (16 to 512 μg/mL). The RBC suspension was then mixed with the drug solutions and incubated at 37 °C for 1 h. PBS and 0.2% Triton X-100 were used as negative and positive controls, respectively. Following incubation, the mixtures were centrifuged at 1000 × g for 5 min. The supernatant was collected, and the absorbance at 570 nm was measured using a TECAN Infinite M Plex microplate reader. All experiments were performed in triplicate, and the hemolysis percentage was calculated relative to the controls.

### 2.6 Cytotoxicity assay

The cytotoxicity of hinokitiol was evaluated using the CCK-8 (Cell Counting Kit-8) assay. MAT-C Cells in the logarithmic growth phase were seeded into 96-well plates at a density of 5 × 10^3^ cells per well. After 24 h of culture for adherence, the medium was replaced with complete medium containing serial concentrations of hinokitiol (0–1,024 μg/mL, with DMSO as the solvent at a final concentration ≤0.5%), and the cells were further incubated for 24 h. Subsequently, 10 μL of CCK-8 working solution was added to each well and incubated for 2 h, protected from light. The absorbance (OD value) at 450 nm was measured using a microplate reader, and cell viability was calculated according to the formula.

### 2.7 Biofilm colony-forming unit (CFU) assay

The inhibitory effect of hinokitiol on the biofilm formation of *S. epidermidis* (ATCC 35984) was quantified using a microtiter plate assay. Briefly, bacterial suspensions in the logarithmic growth phase were adjusted to 1 × 10^6^ CFU/mL in TSB and inoculated into 24-well flat-bottom plates (1 mL/well). hinokitiol was added to achieve final concentrations of 0.5 × MIC, MIC, and 2 × MIC, while an untreated group served as the growth control. After 24 h of incubation, the supernatants were gently aspirated, and the wells were washed three times with PBS to remove non-adherent (planktonic) bacteria. For biofilm eradication assays, pre-formed *S. epidermidis* biofilms were established by incubating bacterial suspensions at 37 °C for 24 h. Following incubation, planktonic bacteria were gently removed by washing three times with sterile PBS, and various concentrations of hinokitiol (0.5 × MIC, MIC, and 2 × MIC) were subsequently added to the adherent biofilm layers.

The adherent biofilm cells were harvested by mechanical scraping followed by ultrasonic dispersion (40 kHz, 5 min) in PBS. The resulting cell suspensions were serially diluted (10^−1^to 10^−5^), and 20 μL aliquots of each dilution were spot plated onto TSA. After incubation at 37 °C for 24 h, colonies were enumerated from plates containing 30–300 CFUs. The results were expressed as the total CFU of adherent bacteria per well.

### 2.8 Checkerboard assay

The checkerboard assay was employed to evaluate the combined antimicrobial activity of hinokitiol with ceftiofur sodium and ceftizoxime sodium, respectively: two-fold serial dilutions of the cephalosporins (0.125 × MIC to 8 × MIC) were arranged vertically in 96-well plates and crossed with horizontally diluted hinokitiol (0.125 × MIC to 8 × MIC) to generate a two-dimensional concentration matrix; each well was inoculated with 100 μL of drug-containing CAMHB broth and 100 μL of bacterial suspension (final inoculum 5 × 10^5^ CFU/mL), incubated at 35 °C for 16–20 h, and the OD₆₀₀ was measured. The combination MIC was defined as the lowest concentration completely inhibiting visible growth, and the fractional inhibitory concentration index (FICI) was calculated to interpret interactions as synergism (FICI ≤ 0.5), additivity (0.5 < FICI ≤ 1.0), indifference (1.0 < FICI ≤ 4.0), or antagonism (FICI > 4.0), with three biological replicates per group.

### 2.9 Crystal violet staining

The inhibitory effect of hinokitiol on biofilm formation was determined using the crystal violet (CV) staining method in 96-well plates. Briefly, *S. epidermidis* suspensions (1 × 10^6^ CFU/mL) were prepared in TSB supplemented with 1% glucose. A 100 μL aliquot of the bacterial suspension was added to each well, followed by the addition of 100 μL of hinokitiol at various concentrations (0.5 × MIC, MIC, and 2 × MIC). Wells containing only TSB and bacteria served as the growth control. After static incubation at 37 °C for 24 h, the culture medium was aspirated, and the wells were gently washed three times with phosphate-buffered saline (PBS) to remove planktonic cells. The remaining biofilms were fixed at 60 °C for 30 min and then stained with 0.1% crystal violet for 15 min. Excess dye was rinsed away with deionized water. Finally, the bound crystal violet was solubilized with 200 μL of 95% ethanol, and the absorbance was measured at 570 nm using a microplate reader. The biofilm inhibition rate was calculated relative to the untreated control.

### 2.10 Assessment of biofilm metabolic activity (XTT assay)

The metabolic activity of *S. epidermidis* biofilms was quantified using the XTT [2,3-bis-(2-methoxy-4-nitro-5-sulfophenyl)-2H-tetrazolium-5-carboxanilide] reduction assay. Following the treatment of biofilms with various concentrations of hinokitiol (0.5 × MIC, MIC, and 2 × MIC) for 24 h, the wells were washed three times with PBS to remove planktonic cells. An XTT-menadione solution was prepared by mixing XTT (1 mg/mL in PBS) with menadione (10 mM in acetone) at a volume ratio of 12.5:1. Each well was then filled with 200 μL of this solution and incubated at 37 °C in the dark for 2–3 h. During this period, the colorless XTT was reduced to an orange formazan product by the mitochondrial dehydrogenases of viable bacteria. Subsequently, 150 μL of the supernatant was transferred to a new 96-well plate, and the absorbance was measured at 470 nm using a microplate reader.

### 2.11 Scanning electron microscopy (SEM) observation

The morphological changes of *S. epidermidis* biofilms treated with hinokitiol were observed using SEM. Briefly, biofilms were grown on sterile glass coverslips (or silicon wafers) placed in 24-well plates and treated with hinokitiol (0.5 × MIC, MIC, and 2 × MIC) for 24 h. After incubation, the coverslips were gently washed three times with PBS to remove planktonic cells. The adherent biofilms were then fixed with 2.5% glutaraldehyde at 4 °Covernight. Following fixation, the samples were dehydrated through a graded series of ethanol (30, 50, 70, 80, 90, and 100%) for 15 min at each step. The dehydrated samples were further dried using a critical point dryer (or hexamethyldisilazane, HMDS) and sputter-coated with gold. Finally, the specimens were examined under a scanning electron microscope to visualize the biofilm architecture and bacterial morphology.

### 2.12 RT-qPCR determination

To investigate the effect of hinokitiol on the transcriptional levels of biofilm-related genes, RT-qPCR was performed. *S. epidermidis* biofilms were grown in 6-well plates and treated with hinokitiol (MIC). Three replicates were set for each treatment group. After the compound incubation, the bacterial solution was centrifuged at 8000 g and washed three times with PBS. The total RNA of the bacteria was extracted using the Tiangen Total RNA Kit. Then the RNA is reverse transcribed into cDNA. Finally, the reaction system was prepared using the SYBR Premix EX Taq II Kit for RT-qPCR detection. The thermal cycling conditions were as follows: initial denaturation at 95 °C for 30 s, followed by 42 cycles of 95 °C for 5 s and 60 °C for 30 s, and a final melting curve analysis (95 °C for 5 s, 60 °C for 1 min, and 97 °C continuous). The data results of RT-qPCR were analyzed using 16S rRNA as the internal reference gene and the relative expression level 2^−∆∆Ct^ method. All experiments were performed independently in triplicate (three biological replicates), and data were presented as mean ± standard deviation (SD). The mRNA primers were synthesized by BGI, Inc. The primer sequences are shown in Table 1.

**Table 1:** Primer sequences of target genes (Cai et al., 2022; Xi et al., 2024).

Primers	Sequence (5′ → 3′)	Tm (°C)	Product size (bp)
icaA-F	CCACGTGCTCTATGCTGGAT	59.89	76
icaA-R	CTTGAGCCCATCGAACCCTT	60.04	
Aap-F	CTTGGACGGCTACGTTATCT	57.13	126
Aap-R	AATCAGCTCTCATAACGCCA	57.01	
icaR-F	TCAAAGCGATGTGCGTAGGA	59.35	193
icaR-R	TCCATTGACGGACTTTACCAGT	59.54	
sigB-F	GAAATCGCACAACGCTTAGA	56.85	83
sigB-R	ACACTCAGGGCATTGTAACT	56.75	
agrA-F	ACGTTCATCAAGCTGTGCTA	57.54	158
agrA-F	TTGAGTTAAAGCGGGGAAGT	57.06	
Luxs-F	GCCTGGTCTACATTCCTT	54.3	145
Luxs-R	CGTCATCGTAGTCGTCAT	54.5	
fnbA-F	GGTGGTGTTGGTGGTACGAT	59.96	161
fnbA-R	TACAAATCCAGGTGGCGGTC	60.04	
sarA-F	AACCTCAAGTTGTTAAAGCTG	54.61	79
sarA-F	CTTTCATCTTTTTCGTTACGTT	53.70	
16S-rRNA-F	GGCAAGCGTTATCCGGAATT	58.5	101
16S-rRNA-R	GTTTCCAATGACCCTCCACG	58.6	

### 2.13 Assessment of bacterial membrane permeability

To assess the impact of hinokitiol on the membrane permeability of *S. epidermidis*, Propidium Iodide (PI) and 1-*N*-phenylnaphthylamine (NPN) uptake assays were performed.

*Staphylococcus epidermidis* cells in the exponential phase were collected, washed, and resuspended in PBS (OD_600nm_ = 0.5). The cells were treated with various concentrations of hinokitiol (0.5 × MIC, MIC, and 2 × MIC) for 2 h at 37 °C. Subsequently, PI was added to a final concentration of 10 μM and incubated in the dark for 15 min. The fluorescence intensity was measured using a fluorescence microplate reader at an excitation wavelength of 535 nm and an emission wavelength of 617 nm.

For the NPN assay, the cell suspension (OD_600nm_ = 0.5) was first incubated with 10 μM NPN at 37 °C for 30 min in the dark. After reaching a stable baseline, hinokitiol was added at the specified concentrations. Membrane hydrophobic permeability was evaluated by adding NPN and recording fluorescence at Ex/Em = 350/420 nm.

Take 1 × 10^8^ CFU/mL of bacterial suspension and transfer it to a sterile 1.5 mL centrifuge tube. Add hinokitiol at concentrations of 0.5 × MIC, MIC, and 2 × MIC, using an equal volume of PBS as a blank control. After incubation at 37 °C for 3 h, add 100 μL of propidium iodide (PI) to a final concentration of 10 μM, followed by 15 min of dark incubation. The fluorescence spectra of the samples were measured using a multifunctional microplate reader with excitation at 530 nm and emission at 570 nm to determine PI fluorescence intensity.

Protein leakage determination: The protein content in the supernatant of *S. epidermidis* cultures treated with hinokitiol was measured by the BCA method. The bacterial liquid in the logarithmic growth phase was washed three times with PBS (pH = 7.2–7.4), and the OD_600_ ratio was adjusted to 0.5. Treatment with hinokitiol (0.5 × MIC, MIC, and 2 × MIC). Incubate for 2 h. Subsequently, centrifuge, take the supernatant, and use the BCA detection kit to detect the protein content in the supernatant. Three parallel tests are set for each test, and each test is repeated three times.

### 2.14 Intracellular pH determination

To evaluate the disruption of the proton motive force, Detection of cell membrane pH gradient (pH): The effect of hinokitiol on the pH of *S. epidermidis* cell membranes was determined using the fluorescent dye BCECF-AM. The OD_600nm_ of the overnight *S. epidermidis* culture was adjusted to 0.5 with PBS. Dilute BCECF-AM (5 mM) in 200 μL opaque 96-well plates and incubate at 37 °C in the dark for 20 min. Then it was treated with hinokitiol (0.5 × MIC, MIC, and 2 × MIC). After incubation for 1 h, the fluorescence values at excitation and emission wavelengths of 488 nm and 535 nm were measured using a multifunctional microplate reader, and the level of pH was calculated.

### 2.15 Bacterial ROS determination

The effect of measuring the ROS level in *S. epidermidis* treated with Hinokitiol using the fluorescent dye DCFH-DA. In simple terms, wash the overnight bacterial culture with PBS (pH = 7.2–7.4), then resuspend it until the OD_600nm_ is 0.5. Then, DCFH-DA with a final concentration of 10 μM was added to the resuscitation solution. Meanwhile, another resuscitation solution was aliquoted into opaque 96-well microplate plates and incubated at 37 °C for 20 min without adding dye. Subsequently, *S. epidermidis* was treated with Hinokitiol (0.5 × MIC, MIC, and 2 × MIC) for 1 h. The ROS levels were measured at an excitation wavelength of 488 nm and an emission wavelength of 525 nm.

### 2.16 RNA extraction and prokaryotic transcriptomic analysis

To further elucidate the molecular mechanism of hinokitiol against *S. epidermidis* biofilms, a prokaryotic transcriptomic analysis was performed. Prokaryotic transcriptomic analysis was conducted using the data generated by the Illumina platform. All analyses were conducted using the cloud platform^1^ of Shanghai Meiji Biomedical Technology Co., LTD. The main software and parameters are as follows: data quality control: The Illumina platform converts the sequencing image signals into text signals through CASAVA base recognition and stores them in fastq format as raw data. Remove the adapter sequence from the reads; Shear and remove the bases at the 5 ‘end that are not A, G, C, or T. Trim the ends of reads with lower sequencing quality (sequencing quality values less than Q20); Remove reads with a proportion of N reaching 10%; Discard the small segments with a length of less than 25 bp after adapter removal and quality pruning. The high-quality reads obtained after the above series of quality shearing are called clean data. Read alignment reference genome: High-quality Reads in each sample are aligned with the reference genome. Analysis tools: Bowtie2^2^. Ribosomal RNA contamination rate assessment: 10,000 original reads were randomly selected from each sample using the blast method and compared with the Rfam database^3^. Based on the annotation results, calculate the percentage of rRNA in each sample and evaluate the rRNA contamination rate.

### 2.17 Statistical analysis

All experiments were performed in at least three independent biological replicates, and the data are presented as the mean ± standard deviation (SD). Statistical analysis was conducted using GraphPad Prism 8.0. Differences between the control and treatment groups were analyzed using one-way analysis of variance (ANOVA). A *p*-value < 0.05 was considered statistically significant (*p < 0.05; **p < 0.01; ***p < 0.001).

## 3 Result

### 3.1 The antibacterial effect of hinokitiol

**Figure 1:**
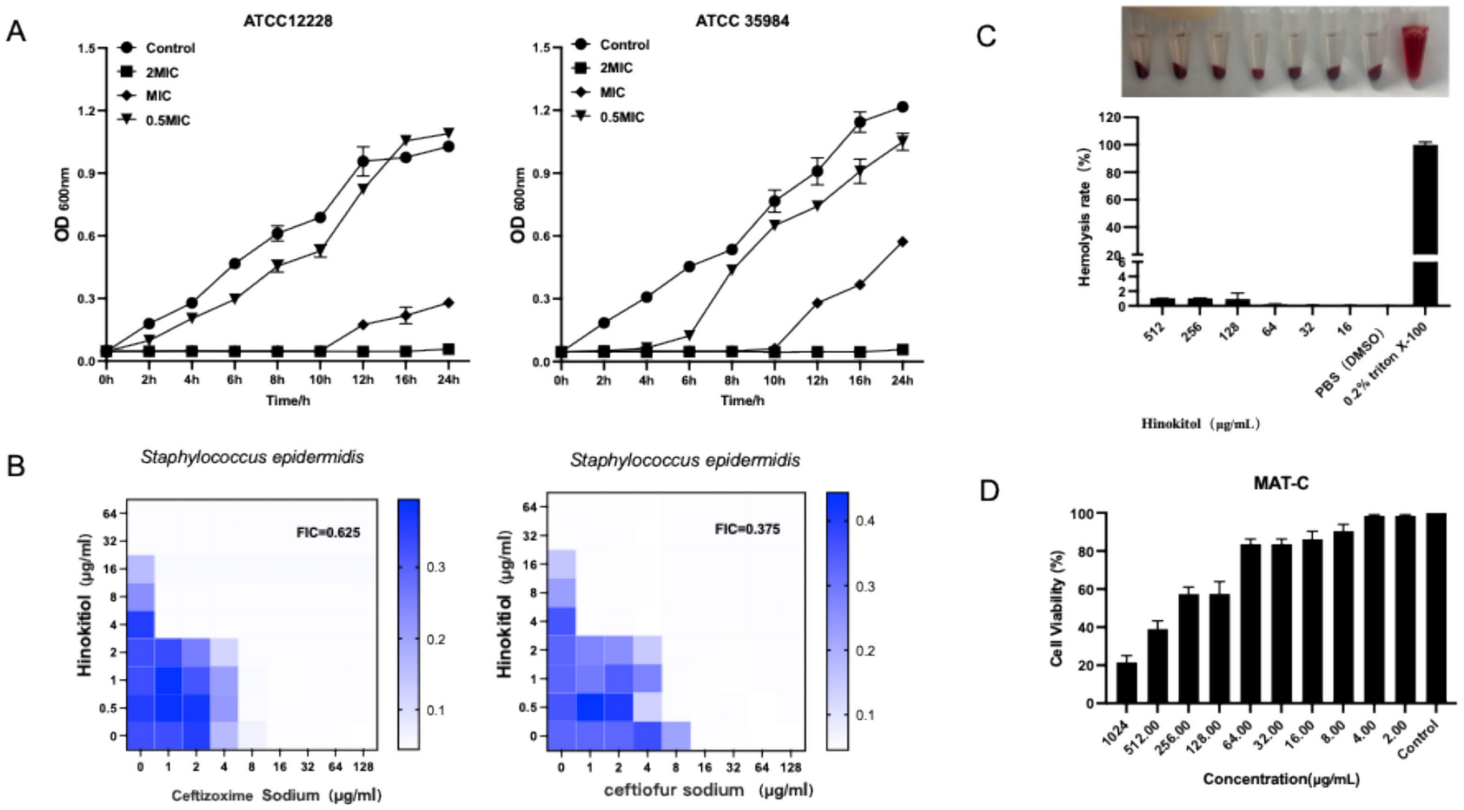
Analysis of antibacterial effect of hinokitiol. **(A)** Determination of the antibacterial curve; **(B)** Combined drug sensitivity test; **(C)** Hemolytic analysis of hinokitiol; **(D)** *In vitro* cytotoxicity of hinokitiol.

To investigate the antibacterial activity of the terpenoid compound hinokitiol against *S. epidermidis*, the minimum inhibitory concentrations (MICs) of several terpenoids against both *S. aureus* and *S. epidermidis* were initially determined (Table 2 and Table 3). The results demonstrated that hinokitiol exhibited significantly stronger antibacterial activity compared to the other tested compounds. As shown in the growth curve in Figure 1A, the control group grew rapidly from 0 to 12 hours and reached the stationary phase after 24 hours. In contrast, the group treated with 2 × MIC (minimum inhibitory concentration) of hinokitiol demonstrated antibacterial effects as early as 2 hours, while the 0.5 × MIC group exhibited inhibition starting at 4–6 hours.

**Table 2:** MIC values of terpenoid compound.

Strain	ATCC-29213 (μg/mL)	ATCC-49576 (μg/mL)	ATCC-12228 (μg/mL)	ATCC-35984 (μg/mL)
α-pinene	>128	>128	>128	>128
(S)- (+)-Carvone	>128	>128	>128	>128
Limonene capsules	>128	>128	>128	>128
Potassium sulfate	>128	>128	>128	>128
Hinokitiol	8	8	8	16
Perillyl alcohol	>128	>128	>128	>128

**Table 3:** MIC detection results of hinokitiol.

*Strain*	*S. aureus* 6#	*S. aureus* 8#	*S. aureus 47#*	*S. haemolyticus 17#*	*S. agalactiae*
Hinokitiol	8	8	8	4	4

To evaluate the potential interaction between hinokitiol and antibiotics (ceftiofur sodium and cefixime sodium), a checkerboard microdilution assay was performed on biofilm-producing *S. epidermidis* strains that are insensitive to these antibiotics. As shown in Figure 1B, the combination of hinokitiol and ceftiofur sodium significantly reduced the individual MICs against *S. epidermidis*: the MIC of Hinokitiol decreased from 16 to 2 μg/mL, and that of ceftiofur sodium dropped from 16 to 4 μg/mL. The fractional inhibitory concentration index (FICI) was calculated to be 0.375. Since the FICI is below 0.5, a strong synergistic effect was confirmed. These results indicate that hinokitiol effectively enhances bacterial susceptibility to antibiotics, likely by compromising the integrity of the bacterial cell wall or membrane, thereby facilitating intracellular antibiotic uptake.

Furthermore, hemolysis assays using sheep blood indicated a favorable safety profile with a low hemolytic rate (Figure 1C). The biocompatibility of hinokitiol was further assessed using the CCK-8 assay with MAT-C cells. As shown in Figure 1D, hinokitiol displayed negligible cytotoxicity at concentrations up to 32 μg/mL, with cell viability remaining above 90%. Although a significant reduction in cell viability was observed at concentrations exceeding 256 μg/mL, the wide safety window suggests that hinokitiol is potentially safe for topical application at its effective antibacterial concentrations.

### 3.2 Hinokitiol destroys the biofilm formation of *Staphylococcus epidermidis*

**Figure 2:**
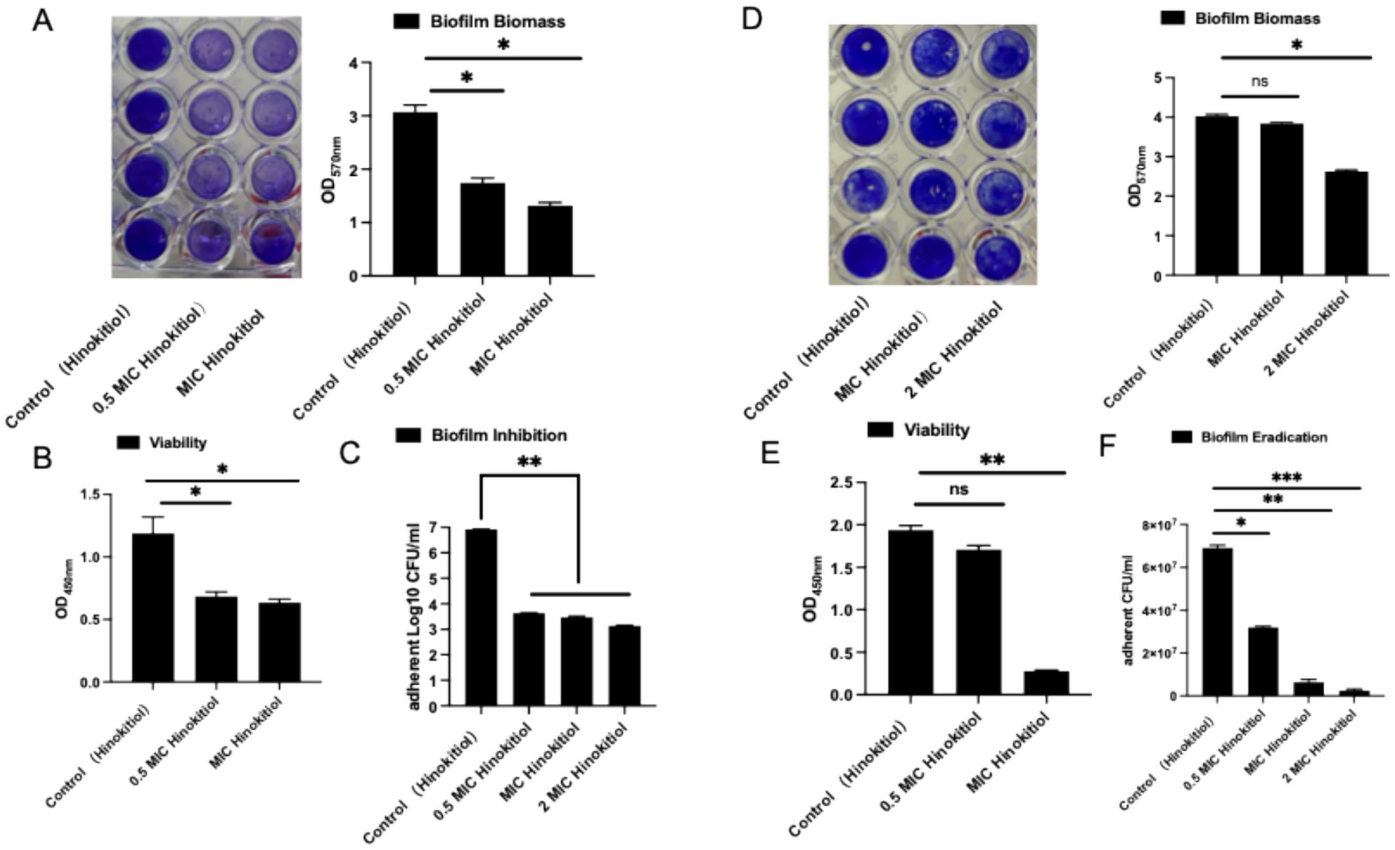
Inhibition and eradication effect of hinokitiol on *S. epidermidis* (ATCC 35984) biofilm formation. **(A,D)** Total biofilm biomass quantified by Crystal Violet staining; **(B,E)** Metabolic activity of sessile cells measured by the XTT assay; **(C,F)** Number of viable sessile cells determined by CFU counting (expressed as Log10 CFU/ml). ns, indicates no significant difference; *, *p* < 0.05, indicates significant difference; **, *p* < 0.01, indicates highly significant difference; ***, *p* < 0.001, indicates extremely significant difference.

To comprehensively evaluate the antibiofilm potential of hinokitiol, we investigated its effects on biofilm biomass, metabolic activity, and the number of viable sessile cells during the formation phase. As shown in Figure 2, hinokitiol exhibited a potent, dose-dependent inhibitory effect on biofilm development. The Crystal Violet (CV) assay demonstrated that hinokitiol dose-dependently inhibited the development of *S. epidermidis* biofilms (Figure 2A). At concentrations of 0.5 × MIC and MIC, the total biomass was reduced. This trend was further supported by the XTT assay, which showed a significant decrease in the metabolic activity of the biofilm-embedded cells (Figure 2B), suggesting a suppression of bacterial fitness during the transition to a sessile lifestyle. To determine whether the reduction in biomass was due to a decrease in the number of culturable cells, adherent colony counting was performed. As shown in Figure 2C, Hinokitiol treatment led to a dramatic, concentration-dependent decline in the number of viable sessile cells. In the control group, the adherent population reached 10^7^ CFU/mL. However, treatment with hinokitiol at 0.5 × MIC and MIC resulted in a reduction in CFU counts, respectively (*p < 0.05*). This significant drop in culturability confirms that hinokitiol effectively prevents the accumulation and survival of *S. epidermidis* on the surface. To further explore whether hinokitiol could eradicate established biofilms of *S. epidermidis*, biofilm-producing strains (ATCC 35984) were incubated for 24 h to form mature biofilms before exposure to various concentrations of hinokitiol. Crystal Violet (CV) staining showed that hinokitiol dose-dependently reduced total biofilm biomass at 0.5 × MIC and MIC concentrations (Figure 2D). As shown in Figure 2E, the XTT assay further confirmed that the metabolic activity of sessile cells was significantly suppressed. Meanwhile, colony counting of adherent bacteria (CFU) demonstrated that hinokitiol treatment resulted in a significant As shown in Figure 2F, concentration-dependent decline in viable cell numbers (decreasing from 10^7^ CFU/mL in controls to lower levels, *p < 0.05*). These consistent results indicate that Hinokitiol not only effectively prevents bacterial colonization and accumulation on surfaces but also eradicates established biofilm structures by compromising bacterial survival.

### 3.3 The effect of hinokitiol on the permeability of *Staphylococcus epidermidis* biofilms

**Figure 3:**
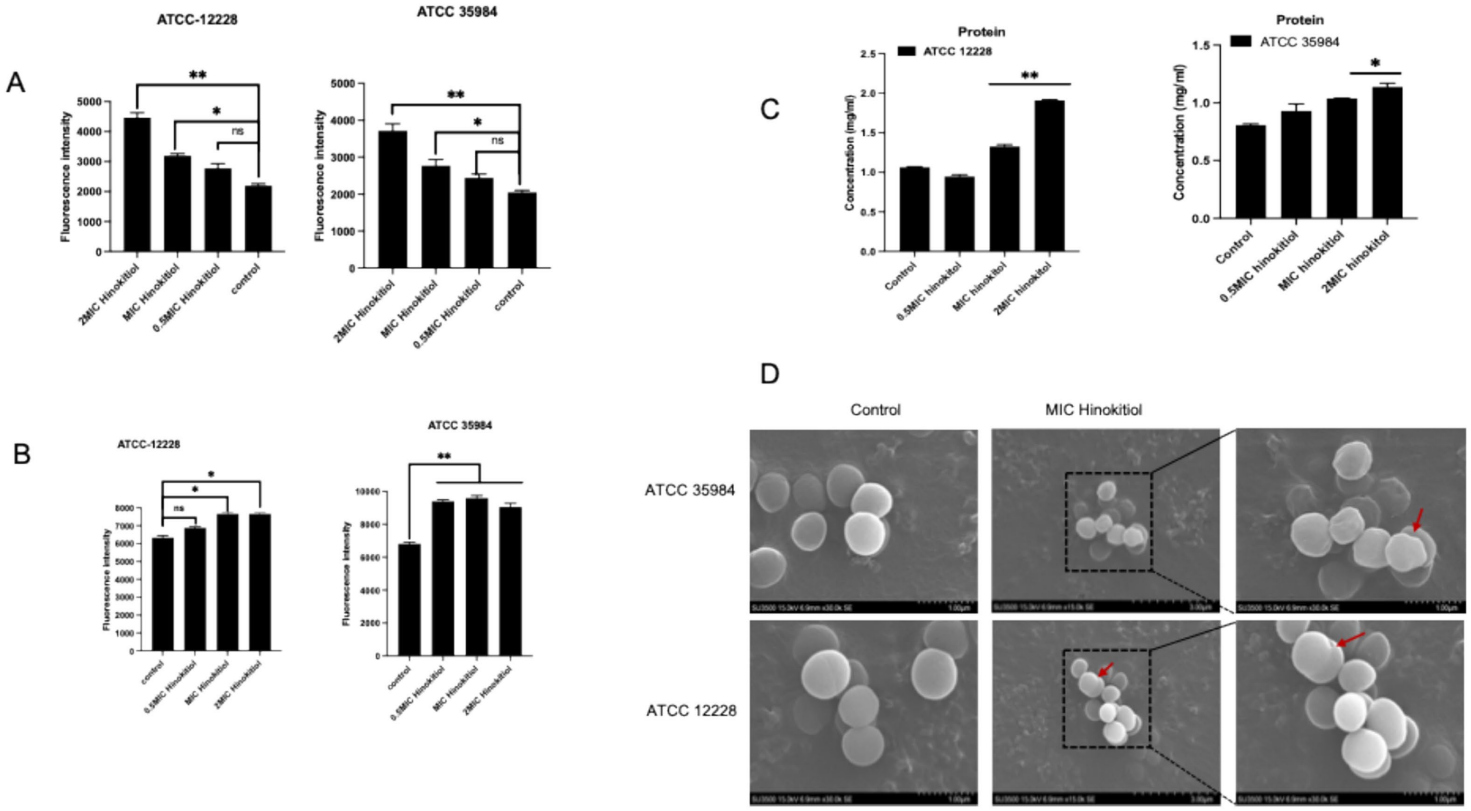
Evaluation of membrane integrity and morphological changes in *S. epidermidis* induced by hinokitiol. **(A)** An assessment of membrane permeability using the fluorescent probe NPN. **(B)** Analysis of cytoplasmic membrane integrity via PI uptake assay. **(C)** Quantification of intracellular protein leakage into the extracellular medium. **(D)** Scanning electron microscopy (SEM) micrographs of *S. epidermidis* (ATCC 12228 and ATCC 35984) treated with different concentrations of hinokitiol for 6 h. Red arrows indicate surface collapse, membrane wrinkling, and structural disintegration. Data are presented as mean ± SD of three independent experiments. *p < 0.05, **p < 0.01.

The structural and functional integrity of the cell membrane is crucial for bacterial survival. The integrity of the cell membrane is evaluated by changes in the permeability of the inner and outer membranes, as well as leakage of bacterial contents. Høiby et al. (2010) effectively adheres to abiotic surfaces and protects against antibiotic penetration. To elucidate the antimicrobial mechanism of hinokitiol, we investigated its impact on the structural and functional integrity of *S. epidermidis* membranes. As shown in Figure 3A, hinokitiol treatment induced a dose-dependent increase in NPN fluorescence intensity, indicating that the drug disrupts the lipid bilayer and significantly enhances membrane permeability. This was further supported by the PI uptake assay (Figure 3B), where a marked elevation in fluorescence was observed, signifying irreversible damage to the cytoplasmic membrane and the formation of transmembrane pores.

The physical breach of the cell membrane led to the leakage of essential intracellular constituents. A significant increase in extracellular protein concentration was detected using the BCA assay following hinokitiol exposure (Figure 3C), confirming the loss of the bacterial permeability barrier. To directly visualize the resulting ultrastructural damage, SEM was employed to characterize the morphology of *S. epidermidis* after 6 h of treatment (Figure 3D). Untreated control cells of both ATCC 12228 and ATCC 35984 exhibited a typical smooth and intact coccoid appearance. In contrast, cells treated with hinokitiol showed severe morphological abnormalities, including irregular cell shapes, surface corrugation, and deep structural collapse (highlighted by red arrows). Notably, in the biofilm-forming strain ATCC 35984, the treatment not only damaged individual cells but also appeared to disintegrate the protective extracellular matrix. In conclusion, these findings demonstrate that hinokitiol exerts its bactericidal activity by physically disrupting the cell membrane and biofilm architecture.

### 3.4 Transcriptomic analysis of hinokitiol treatment on the *Staphylococcus epidermidis*

**Figure 4:**
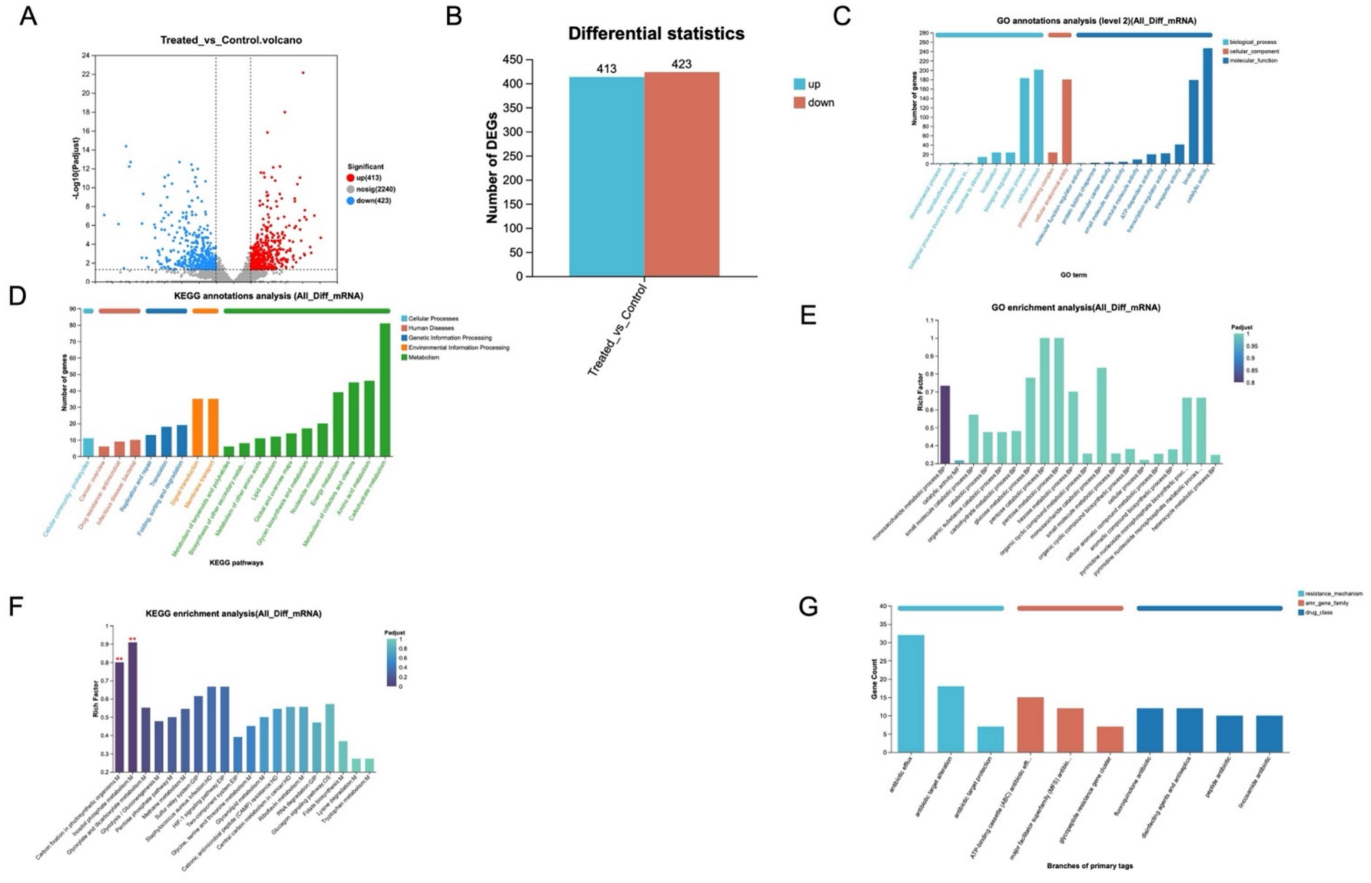
Prokaryotic transcriptomic analysis. **(A)** Large volcano map of differentially expressed genes; **(B)** The number of differentially expressed genes; **(C)** GO annotation analysis; **(D)** KEGG annotation analysis; **(E)** GO enricher analysis. **(F)** KEGG enricher analysis; **(G)** CARD annotation analysis.

To evaluate the effect of Hinokitiol on the bacterial transcriptome, RNA-Seq analysis was performed on three biological samples obtained after treating *S. epidermidis* with Hinokitiol and a blank control. Through analysis, it can be seen whether there are differences between sample groups or the consistency of the samples. It can be seen from the analysis results that the distribution of the Hinokitiol treatment group (Treated) and the Control group (Control) is obvious, indicating that there is a significant difference between the Treated and Control groups, and the differences among each group are remarkable. Expression level difference analysis is the analysis of the differential expression of genes among samples to identify the differentially expressed genes among samples. For experimental designs with biological duplication, this study directly used the DESeq2 software based on the negative binomial distribution to statistically analyze raw counts. Differentially expressed genes were obtained based on certain screening conditions, with the default parameters being *p < 0.05*& log^2^FC ≥ 1. The results are shown in Figure 4.

There is a total of 836 differentially expressed genes, among which 413 are up-regulated, and 423 are down-regulated (Figures 4A,B). The COG data annotation results indicated that differentially expressed genes (DEGs) were mainly related to cellular processes and signal transduction, as well as metabolism. Among them, 67 genes were involved in translation, ribosome structure and biogenesis, 33 genes were involved in the formation of cell walls/membranes/envelopes, and 35 genes were involved in inorganic ion transport and metabolism (Figure 4C). KEGG pathway analysis revealed that DEGs were mainly involved in metabolic pathways such as carbohydrate metabolism, energy metabolism, amino acid metabolism, and signal transduction. Among them, the external environmental processes were mainly signal transduction and membrane transport (Figure 4D). GO and KEGG enrichment analysis determined that DEGs were mainly enriched in cellular process metabolic processes, protein-containing complexes, and transport activities. Among them, the biological process is mainly cellular metabolism, and the molecular functions are mainly reflected in protein transport and catalytic functions (Figures 4E,F). In addition, the CARD annotation mainly explains that it may be related to the antibiotic efflux pump and ATP-binding cassette transporters, and the transporter superfamily (MFS) antibiotic efflux pump, providing ideas for our subsequent research (Figure 4G). In summary, we have found that the differential genes are mainly enriched in the ABC transport system, which is mainly reflected in the uptake of nutrients, drug excretion, secretion of virulence factors, and the formation of biofilms. Studies have shown that the ABC transport system may also play a role in the formation and maintenance of bacterial biofilms. Therefore, it is necessary to further explore whether hinokitiol can inhibit the expression of genes that form biofilms, thereby influencing the quorum-sensing system and suppressing the formation of biofilms.

### 3.5 The effect of hinokitiol on the functional integrity of the biofilm of *Staphylococcus epidermidis*

**Figure 5:**
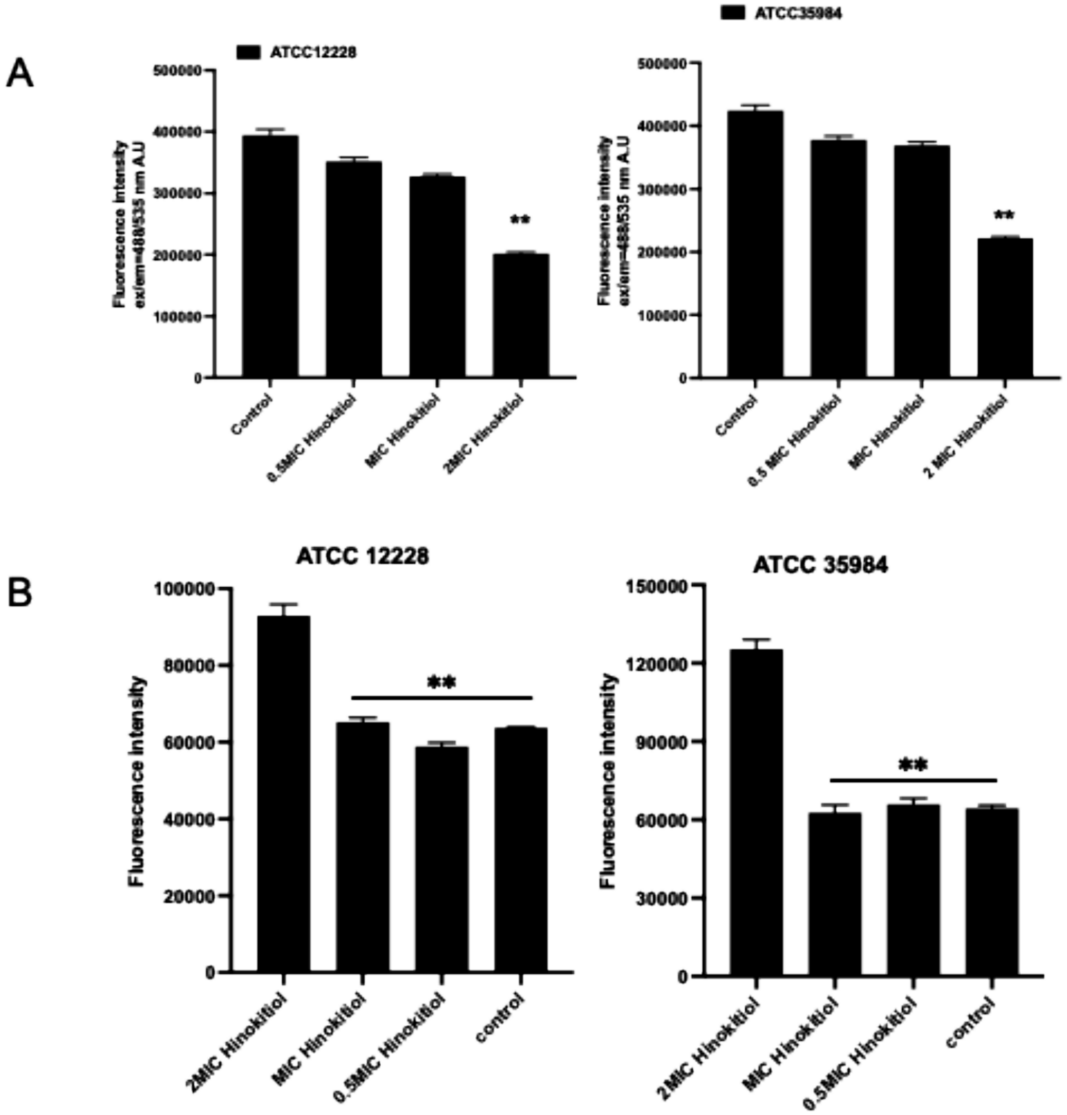
Determination of cell membrane functional integrity of *S. epidermidis*. **(A)** The effect of Hinokitiol treatment on pH of *S. epidermidis*; **(B)** The effect of Hinokitiol treatment on ROS in *S. epidermidis*. ns, indicates no significant difference; *, *p* < 0.05, indicates significant difference; **, *p* < 0.01, indicates highly significant difference; ***, *p* < 0.001, indicates extremely significant difference.

Reactive oxygen species (ROS) are natural by-products of cellular oxidative metabolism. Under physiological conditions, ROS levels are tightly regulated; however, excessive intracellular accumulation of ROS induces oxidative stress (Zheng et al., 2025). To investigate the impact of hinokitiol on bacterial bioenergetics and redox homeostasis, we measured the transmembrane pH gradient (pH) and intracellular reactive oxygen species (ROS) levels in *S. epidermidis* (ATCC 12228and ATCC 35985). As shown in Figure 5A, hinokitiol induced a concentration-dependent decrease in the fluorescence intensity of the pH-sensitive probe, indicating a significant dissipation of ΔpH compared to the untreated control. This effect was evident even at sub-inhibitory concentrations, suggesting that the disruption of proton homeostasis is a primary event in hinokitiol’s antibacterial action. Furthermore, ROS levels were quantified using the DCFH-DA fluorescent probe (Figure 5B). Treatment with hinokitiol at higher concentrations (0.5 × MIC, MIC, and 2 MIC) triggered a significant elevation in intracellular ROS levels (*p* < 0.05). Interestingly, no statistically significant increase in ROS was observed at lower doses, implying that oxidative stress-mediated damage may be a secondary or concentration-dependent mechanism that complements the direct disruption of membrane-associated functions. The occurrence of severe oxidative damage in biofilm-forming strains, mirroring the trends seen in non-biofilm-forming counterparts, provides compelling evidence that hinokitiol can penetrate the protective matrix to significantly disrupt biofilm formation.

### 3.6 Hinokitiol regulates the gene expression of biofilms and inhibits the formation of biofilms

**Figure 6:**
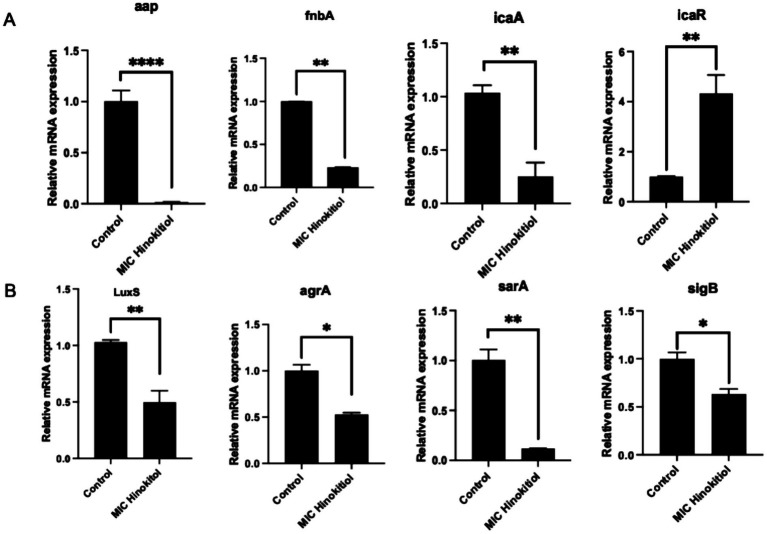
Effects of hinokitiol on the transcript levels of biofilm- and virulence-related genes in *S. epidermidis*. **(A)** The relative mRNA expression levels of genes involved in biofilm formation (aap, icaA, icaR, fnbA) were determined by RT-qPCR after treatment with Hinokitiol at different concentrations (MIC) for 6 h; **(B)** Quorum sensing (luxS, agrA), and global regulation (sarA, sigB) were determined by RT-qPCR after treatment with hinokitiol (MIC) for 12 h. The 16S rRNA gene was used as an internal control for normalization. Data are presented as the mean ± SD of three independent experiments. Statistical significance was analyzed using one-way ANOVA: *p < 0.05, **p < 0.01, and ***p < 0.001. ns, indicates no significant difference; *, *p* < 0.05, indicates significant difference; **, *p* < 0.01, indicates highly significant difference; ***, *p* < 0.001, indicates extremely significant difference.****, *p* < 0.0001, indicates an extraordinarily significant difference.

The biofilm of *S. epidermidis* is closely related to pathogenicity and is regulated and synthesized by the icaADBC operon. The transcription factor SigB can regulate the expression of icaADBC through unknown factors (Hoang et al., 2019). By investigating the regulation of the global regulator sarA by sigB, as well as the subsequent regulation of icaADBC by SarA, this study sought to clarify whether sigB modulates icaADBC expression through *sarA* to control biofilm formation. To further explore the effect of hinokitiol on the mechanism of biofilm formation and to elucidate the potential inhibitory mechanism, we used RT-qPCR to analyze the transcriptional levels of biofilm-related genes in hinokitiol-treated and untreated *S. epidermidis* isolates (Figures 6A,B). After treatment with hinokitiol at the MIC, the expression levels of *aap, sarA, icaA, agrA, fnbA, sigB,* and *luxS* genes were down-regulated, while the expression level of the repressor icaR was up-regulated. These transcriptional changes corroborate the observed phenotypic alterations in biofilms and further reveal the mechanism by which hinokitiol inhibits the adhesion of *S. epidermidis* and the production of Polysaccharide Intercellular Adhesin (PIA).

To further investigate the molecular inhibitory mechanism of hinokitiol on biofilm formation and virulence, the transcript levels of several key genes were quantified by RT-qPCR. The results demonstrated that hinokitiol treatment significantly modulated the expression of genes involved in intercellular adhesion, Quorum Sensing (QS), and global regulation (Figure 6). Regarding biofilm development, hinokitiol treatment led to a dramatic down-regulation of i*caA, fnbA,* and *aap*, which are essential for PIA production and primary surface attachment, respectively. Interestingly, the expression of icaR, a transcriptional repressor of the ica operon, was significantly up-regulated. This suggests that hinokitiol inhibits biofilm formation by suppressing the ica-dependent pathway and reducing the production of surface-associated proteins. The Quorum Sensing systems of *S. epidermidis* were also profoundly affected. The expression levels of agrA (the response regulator of the agr system) and luxS (involved in AI-2 signaling) were significantly reduced following hinokitiol exposure. Since the agr and luxS systems play pivotal roles in coordinating the expression of virulence factors and biofilm maturation, their down-regulation indicates a comprehensive disruption of bacterial communication and population-level coordination.

Furthermore, the mRNA levels of global regulators sarA and sigB were markedly suppressed. Given that sarA is a positive regulator of the ica operon and various adhesins, its down-regulation is consistent with the observed decrease in biofilm-related gene expression. Similarly, the reduction in *sigB* expression suggests an impaired stress response and a diminished ability of the bacteria to adapt to the antimicrobial pressure exerted by hinokitiol. In summary, the RT-qPCR results were highly consistent with the transcriptomic data. The simultaneous suppression of adhesion-related genes (*icaA, fnbA, aap*), QS components (*agrA, luxS*), and global regulators (*sarA, sigB*) provides strong evidence that hinokitiol exerts its anti-biofilm effect through a multi-pathway regulatory network, effectively preventing the transition from a planktonic to a sessile lifestyle.

## 4 Conclusion

In summary, our *in vitro* findings demonstrate that hinokitiol exhibits effective antibacterial and anti-biofilm activities against *S. epidermidis*, showing a significant synergistic effect with Ceftiofur sodium. Its multi-target mechanism involves disrupting cell membrane integrity, inducing oxidative stress, and inhibiting biofilm-associated regulatory pathways (icaA, agrA, and sarA). These results suggest hinokitiol as a promising candidate for developing novel strategies against *S. epidermidis*-induced infections, particularly in the context of biofilm-associated bovine mastitis. However, further investigations, including comprehensive *in vivo* efficacy and safety assessments, as well as pharmacokinetic studies, are essential to fully validate these in vitro findings and facilitate their translation into practical clinical applications.

## 5 Discussion

This study systematically evaluated the antimicrobial and anti-biofilm activities of the natural monoterpene hinokitiol against *Staphylococcus epidermidis* isolates derived from bovine mastitis. Our findings demonstrate that hinokitiol significantly inhibits biofilm formation at sub-minimum inhibitory concentrations (sub-MIC) while exhibiting favorable biocompatibility with bovine mammary epithelial cells (MAC-T). These results hold significant clinical potential for addressing the increasing prevalence of multidrug-resistant strains, such as methicillin-resistant coagulase-negative staphylococci (MRCNS), in the dairy industry.

As an important opportunistic pathogen causing bovine mastitis, the pathogenicity of *S. epidermidis* largely depends on its colonization of mammary tissue surfaces and biofilm formation. Notably, minimal genetic overlap exists between milk-derived *S. epidermidis* and human clinical strains, indicating that bovine-source isolates possess high host adaptation (Badshah et al., 2025; Kløve et al., 2025). The biofilm-producing strain ATCC35984 selected in this study exhibited exceptional biofilm-forming capacity, and the significant inhibitory effect of hinokitiol on its biofilm further confirms the potential of this natural product in addressing bovine-specific infections. This finding aligns well with the strategy proposed by previous studies (Silva et al., 2025; Sindhu et al., 2025) regarding the use of natural biomolecules, such as flavonoids (Haj Hasan et al., 2024), as alternatives to traditional antibiotics for treating bovine mastitis (Fan et al., 2025). Importantly, these findings align with and extend previous mechanistic studies on natural antibiofilm agents. While quercetin targets SarA/AgrA regulatory proteins (Liu et al., 2024) and andrographolide suppresses adhesion genes (*atlE, aap*) (Zhang et al., 2024), hinokitiol uniquely combines transcriptional repression of matrix synthesis with QS disruption. Similarly, baicalein, a representative flavonoid green biomolecule, has demonstrated significant therapeutic efficacy in MRSA murine mastitis models through multi-target antibacterial mechanisms (Soni et al., 2024). These studies collectively support the strategy as a viable alternative to traditional antibiotics for treating bovine mastitis.

The antibiofilm mechanism of hinokitiol involves a dual-pathway process comprising physical disruption and molecular regulation. Firstly, at the physical level, propidium iodide (PI) staining and reactive oxygen species (ROS) detection demonstrated that hinokitiol induced explosive intracellular ROS accumulation and compromised cell membrane integrity. This effect is attributed to the exceptional lipophilicity of hinokitiol, enabling it to penetrate the polysaccharide matrix of the biofilm and insert into the bacterial lipid bilayer. Wyżga et al. (2024) confirmed through biophysical modeling that hinokitiol causes lipid membrane expansion; the observed decrease in membrane potential and morphological shrinkage in this study aligns with their conclusions. Such physical damage not only directly compromises bacterial viability but may also enhance membrane permeability, generating a bactericidal effect analogous to neutrophil oxidative stress. At the molecular regulatory level, this study elucidated a sophisticated dual-target mechanism by which hinokitiol disrupts *S. epidermidis* biofilm architecture. First, at the transcriptional level, our RT-qPCR results revealed that hinokitiol significantly upregulated the expression of icaR, a negative regulatory factor that strictly governs the *icaADBC* operon. This transcriptional reprogramming consequently downregulated *icaA* expression, thereby fundamentally blocking the synthesis of polysaccharide intercellular adhesin (PIA)—the critical extracellular matrix component for biofilm scaffold construction. This precision-guided regulatory strategy effectively starves the biofilm of its structural foundation at the source.

Furthermore, the quorum-sensing (QS) system serves as a commander in coordinating bacterial collective behaviors. This study revealed that hinokitiol significantly reduced LuxS protein expression levels. As the core enzyme for AI-2 signal molecule synthesis, the decrease in LuxS protein disrupts interbacterial communication, preventing bacteria from synchronously regulating biofilm maturation and dispersal. *S. aureus* has evolved a complex regulatory network that precisely controls biofilm development when the environment changes (Schilcher and Horswill, 2020). In *S. epidermidis*, mutations in rsbU and *σ*B lead to a reduction in biofilm formation. The σ factor SigB regulates genes involved in the formation of various types of biological membranes, including those mainly composed of proteins, extracellular DNA, and PIA. This phenotype is caused by the upregulation of IcaR and the subsequent downregulation of the ica operon (Schilcher and Horswill, 2020). The PIA synthesis and regulation of the ica manipulator are controlled by a complex regulatory network, the details of which are not yet fully understood. The icaADBC manipulator is negatively regulated by *icaR* and *TcaR* (Jefferson et al., 2004). IcaR is a DNA-binding protein (Jefferson et al., 2003) located upstream of the icaADBC operon and serves as a repressor of icaA transcription in *S. epidermidis* (Conlon et al., 2002) and *S. aureus* (Jefferson et al., 2004). The AI-2 (LuxS/autoinducer-2) system was initially discovered in Vibrio Halvibrio (Bassler et al., 1994). LuxS synthesizes AI-2, an S-adenosylmethionine, as the extracellular signaling molecule of this system. Studies have shown that *LuxS* is a negative regulatory factor for biofilm formation (Schauder et al., 2001). These results are attributed to the activation of *icaR* transcription by AI-2, which leads to the downregulation of *icaA* expression (Yu et al., 2012; Schilcher and Horswill, 2020). Sang Research has revealed the interaction between quercetin and quorum sensing regulatory proteins *sarA* and *agrA*, which can effectively inhibit the formation of biofilms, significantly reduce the number of live bacteria in biofilms, and lower the content of matrix components such as extracellular polysaccharides, proteins, and extracellular DNA (Goswami et al., 2025) Diclofenac sodium hinders the formation of *S. epidermidis* biofilms by inhibiting bacterial adhesion and PIA synthesis. Andrographolide can inhibit the formation of biofilms in *S. aureus* (Xi et al., 2024). The mechanism may be by suppressing the expression of key genes such as *icaA, atlE, aap*, and l*uxS* in *S. aureus*, thereby affecting the adhesion, aggregation, and maturation of biofilms (Zhang et al., 2024).

Furthermore, the therapeutic paradigm for mastitis requires addressing both the pathogen and the host inflammatory response. Unlike diflunisal, which attenuates acute inflammatory responses primarily through inhibition of the NF-κB signaling pathway in *S. aureus* -induced mastitis models (Soni et al., 2025), hinokitiol exerts its protective effect by directly dismantling bacterial biofilm structures and eliminating the infection source, thereby reducing inflammatory stimuli at their origin. This distinction highlights the complementary roles of anti-biofilm agents (targeting bacterial persistence) and anti-inflammatory drugs (modulating host immunity) in comprehensive mastitis management. The combination of Hinokitiol’s direct antibacterial action with diflunisal’s inflammation-modulating capacity could represent a promising dual-therapy approach for persistent MRCNS infections, simultaneously eradicating biofilm-embedded pathogens and mitigating tissue damage. Collectively, by simultaneously targeting the *icaR-icaADBC* axis for structural inhibition and the luxS/AI-2 system for behavioral disruption, hinokitiol establishes a comprehensive antivirulence strategy. This multi-pronged approach, which disarms bacteria without exerting lethal selective pressure, provides novel insights for developing next-generation therapeutics against MRCNS-associated persistent infections in dairy cattle.

Despite these promising *in vitro* findings, several limitations must be acknowledged to facilitate clinical translation. First, in vitro models do not fully replicate the complex host-pathogen interactions or the unique microenvironment of the bovine mammary gland, such as the potential interference of milk components with drug activity. As emphasized in recent studies (Soni et al., 2024), translating in vitro efficacy to *in vivo* outcomes remains a major challenge. Second, the absence of comprehensive pharmacokinetic (PK) data in this study limits our ability to determine optimal dosage regimens or predict drug distribution in cattle. Therefore, future research must prioritize establishing relevant in vivo mastitis models to comprehensively evaluate hinokitiol’s efficacy in reducing somatic cell counts (SCC), eliminating intramammary infection, and assessing potential adverse effects. Furthermore, clinical translation will require the development of specialized delivery systems—such as nanocarriers, hydrogels, or liposomal formulations—to improve bioavailability, extend residence time in the mammary gland, and minimize tissue irritation. A critical discussion on the most suitable administration routes, whether local intramammary infusion or systemic delivery, along with optimized formulation strategies, is imperative for advancing hinokitiol towards practical clinical use in bovine mastitis cases. Such advancements are vital for optimizing hinokitiol for field-level treatment protocols.

In summary, it is indicated that the search for effective natural compounds can inhibit the formation of bacterial biofilms, destroy their effective bacterial barriers, and, when combined with antibiotic treatment, can effectively reduce the development of drug resistance. The terpene compound hinokitiol exhibits certain antibacterial effects and is safe, providing a theoretical basis for subsequent treatment of *S. epidermidis.*

## Data Availability

The RNA-seq data generated in this study are publicly available in the Sequence Read Archive (SRA) under BioProject PRJNA1462149. Project information will be accessible at the following link: http://www.ncbi.nlm.nih.gov/bioproject/1462149. Further inquiries can be directed to the corresponding author.

## Footnotes

1
cloud.majorbio.com

2
http://bowtie-bio.sourceforge.net/bowtie2/index.shtml

3
http://rfam.xfam.org/
