# Supplementary material for: Study on the mechanism of hinokitiol in inhibiting the biofilm activity of Staphylococcus epidermidis
Source: Front Microbiol. 2026 May 21;17:1811623. doi: 10.3389/fmicb.2026.1811623 (PMC13233496; doi:10.3389/fmicb.2026.1811623)
Supplement: Supplementary file 1 [file Data_Sheet_1.PDF]

Supplementary Material

1 Supplementary Data

Adhesion and aggregation are crucial for biofilm formation in *Staphylococcus epidermidis*, which specifically reacts with Congo red medium to turn black. As shown in Supplementary Figure 1, increasing hinokitiol concentrations inhibit extracellular polysaccharide PIA synthesis in BF, thereby reducing intercellular adhesion. This is consistent with the RT-qPCR results. After hinokitiol treatment of ATCC 35984, the expression levels of PIA synthesis structural genes *(icaA, icaD, icaC*, and *icaB)* were downregulated, while the negative regulatory factor icaR was upregulated, and global regulatory factors *sarA* and *agrA* were downregulated. These findings indicate that hinokitiol inhibits biofilm formation capability.

Amplification primer sequence:

27F AGTTTGATCMTGGCTCAG 1500bp；1492R GGTTACCTTGTTACGACTT 1500bp.

S1 Sanger 测序。

>rRNA_Chromosome1_2841450-2842990_DIR-/molecule=16s_rRNA /score=2030.9

AGAGTTTGATCCTGGCTCAGGATGAACGCTGGCGGCGTGCCTAATACATGCAAGTCGAG
C

GAACAGATGAGAAGCTTGCTTCTCTGATGTTAGCGGCGGACGGGTGAGTAACACGTGGG
T

AACCTACCTATAAGACTGGGATAACTCCGGGAAACCGGGGCTAATACCGGATAATATTT
T

GAACCGCATGGTTCAATAGTGAAAGACGGTTTCGGCTGTCACTTATAGATGGACCCGCG
C

CGTATTAGCTAGTTGGTAAGGTAACGGCTTACCAAGGCGACGATACGTAGCCGACCTGA
G

AGGGTGATCGGCCACACTGGAACTGAGACACGGTCCAGACTCCTACGGGAGGCAGCAG
TA

GGGAATCTTCCGCAATGGGCGAAAGCCTGACGGAGCAACGCCGCGTGAGTGATGAAGG
TC

TTCGGATCGTAAAACTCTGTTGTTAGGGAAGAACAAATTTGTTAGTAACTGAACAAGTC
T

TGACGGTACCTAACCAGAAAGCCACGGCTAACTACGTGCCAGCAGCCGCGGTAATACGT
A

GGTGGCAAGCGTTATCCGGAATTATTGGGCGTAAAGCGCGCGTAGGCGGTTTCTTAAGT
C

TGATGTGAAAGCCCACGGCTCAACCGTGGAGGGTCATTGGAAACTGGGAAACTTGAGTG
C

AGAAGAGGAGAGTGGAATTCCATGTGTAGCGGTGAAATGCGCAGAGATATGGAGGAAC
AC

CAGTGGCGAAGGCGGCTCTCTGGTCTGTAACTGACGCTGATGTGCGAAAGCGTGGGGAT
C

AAACAGGATTAGATACCCTGGTAGTCCACGCCGTAAACGATGAGTGCTAAGTGTTAGGG
G

GTTTCCGCCCCTTAGTGCTGCAGCTAACGCATTAAGCACTCCGCCTGGGGAGTACGACC
G

CAAGGTTGAAACTCAAAGGAATTGACGGGGACCCGCACAAGCGGTGGAGCATGTGGTT
TA

ATTCGAAGCAACGCGAAGAACCTTACCAAATCTTGACATCCTTTGACCGCTCTAGAGAT
A

GAGTCTTCCCCTTCGGGGGACAAAGTGACAGGTGGTGCATGGTTGTCGTCAGCTCGTGT
C

GTGAGATGTTGGGTTAAGTCCCGCAACGAGCGCAACCCTTAAGCTTAGTTGCCATCATT
A

AGTTGGGCACTCTAGGTTGACTGCCGGTGACAAACCGGAGGAAGGTGGGGATGACGTC
AA

ATCATCATGCCCCTTATGATTTGGGCTACACACGTGCTACAATGGATAATACAAAGGGC
A

GCGAATCCGCGAGGCCAAGCAAATCCCATAAAATTATTCTCAGTTCGGATTGTAGTCTG
C

AACTCGACTACATGAAGCTGGAATCGCTAGTAATCGTAGATCAGCATGCTACGGTGAAT
A

CGTTCCCGGGTCTTGTACACACCGCCCGTCACACCACGAGAGTTTGTAACACCCGAAGC
C

GGTGGAGTAACCTTTTAGGAGCTAGCCGTCGAAGGTGGGACAAATGATTGGGGTGAAGT
C

GTAACAAGGTAGCCGTATCGGAAGGTGCGGCTGGATCACCT

Supplementary Material should be uploaded separately on submission. Please include any supplementary data, figures and/or tables.

Supplementary material is not typeset so please ensure that all information is clearly presented, the appropriate caption is included in the file and not in the manuscript, and that the style conforms to the rest of the article.

2 Supplementary Figures and Tables

2.1 Supplementary Figures

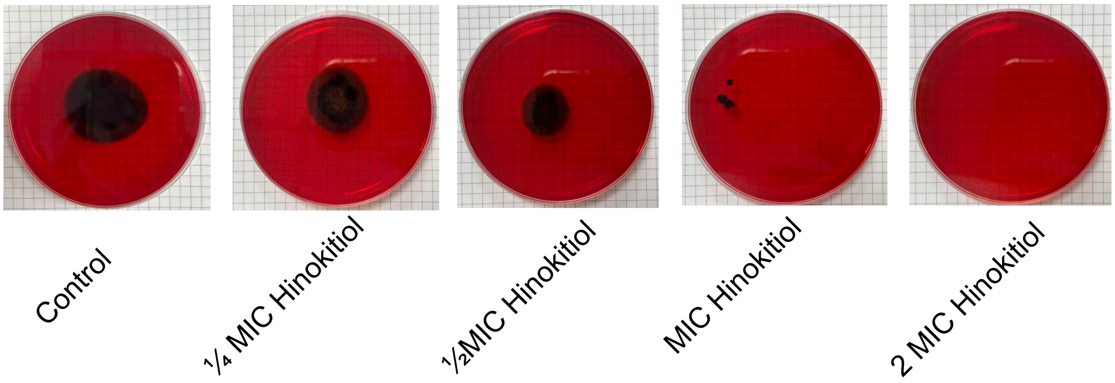

Supplementary Figure 1. Hinokitiol inhibits PIA synthesis in biofilm-forming Staphylococcus epidermidis.

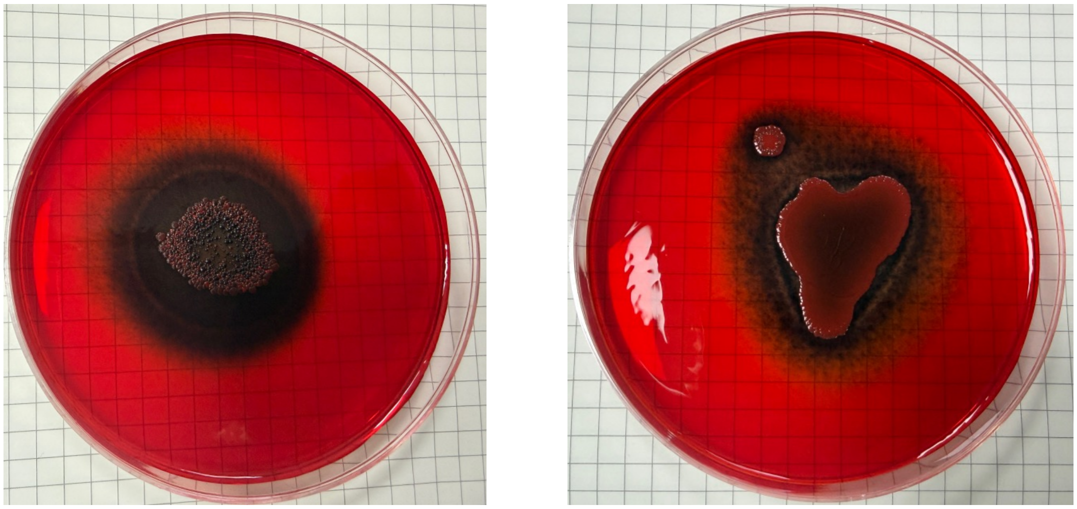

ATCC 35984 ATCC 12228

Supplementary Figure 2. ATCC 12228 is a negative membrane-producing strain.
